# Supplementary material for: Nrp1 is Activated by Konjac Ceramide Binding-Induced Structural Rigidification of the a1a2 Domain
Source: Cells. 2020 Feb 24;9(2):517. doi: 10.3390/cells9020517 (PMC7072815; doi:10.3390/cells9020517)
Supplement: Supplementary file 1 [file cells-09-00517-s001.pdf]

Article

Nrp1 is activated by kCer binding-induced structural rigidification of the a1a2 domain

Seigo Usuki[1*], Yoshiaki Yasutake[2,3], Noriko Tamura[2], Tomohiro Tamura[2,3], Kunikazu Tanji[4], Takashi Saitoh[5], Yuta Murai[6], Daisuke Mikami[1], Kohei Yuyama[1], Kenji Monde[,6], Katsuyuki Mukai[1,7], Yasuyuki Igarashi[1]

[1]Lipid Biofunction Section, Faculty of Advanced Life Science, Hokkaido University, Sapporo, Hokkaido, Japan

[2]Bioproduction Research Institute, National Institute of Advanced Industrial Science and Technology (AIST), Sapporo, Hokkaido, Japan

[3]Computational Bio Big-Data Open Innovation Laboratory (CBBD-OIL), AIST, Tokyo, Japan

[4]Department of Neuropathology, Institute of Brain Science, Hirosaki University Graduate School of Medicine, Hirosaki, Aomori, Japan

[5]Department of Medicinal Chemistry, Faculty of Pharmaceutical Sciences, Hokkaido University of Science, Sapporo, Hokkaido, Japan

[6]Faculty of Advanced Life Science, Hokkaido University, Sapporo, Hokkaido, Japan

[7]R&D Headquarters, Daicel Corporation, Tokyo, Japan

[*]Correspondence: susuki@sci.hokudai.ac.jp Tel: +81-11-706-9086; Fax: +81-11-706-9024

Supplementary Material

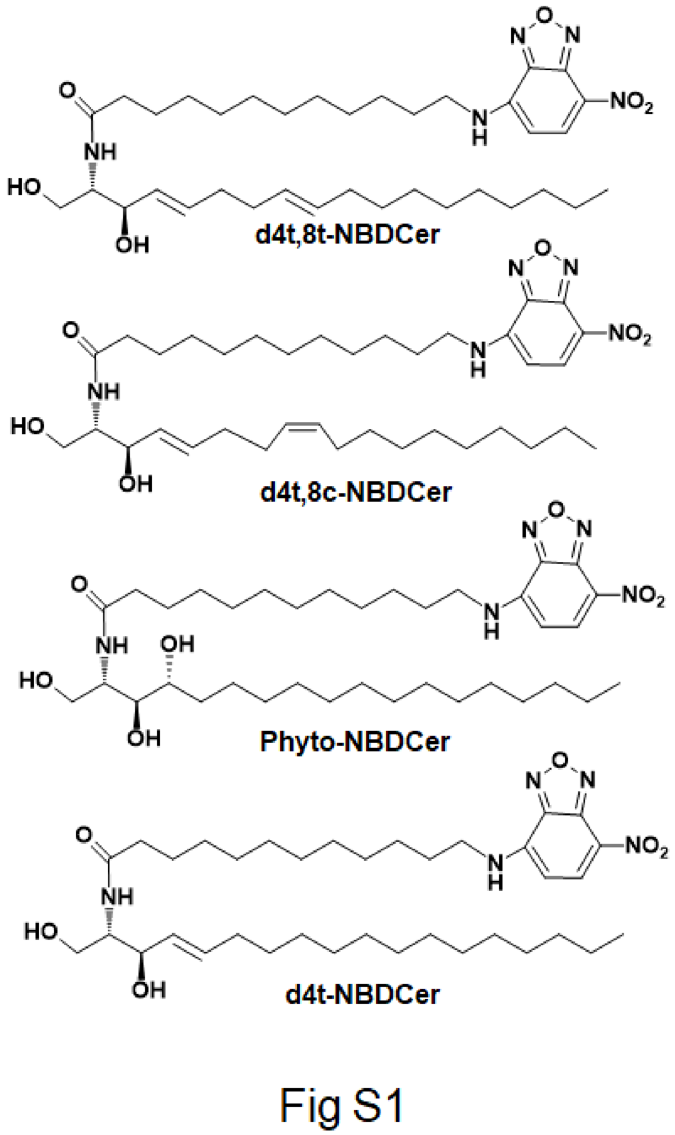

Figure S1: Long-chain bases for d18:2$^{4t, 8t}$, d18:2$^{4t, 8c}$, d18:1^{4t}, and t18:0 (shown in Figure 1A).

Those were acylated by NBD-dodecanoic acid.

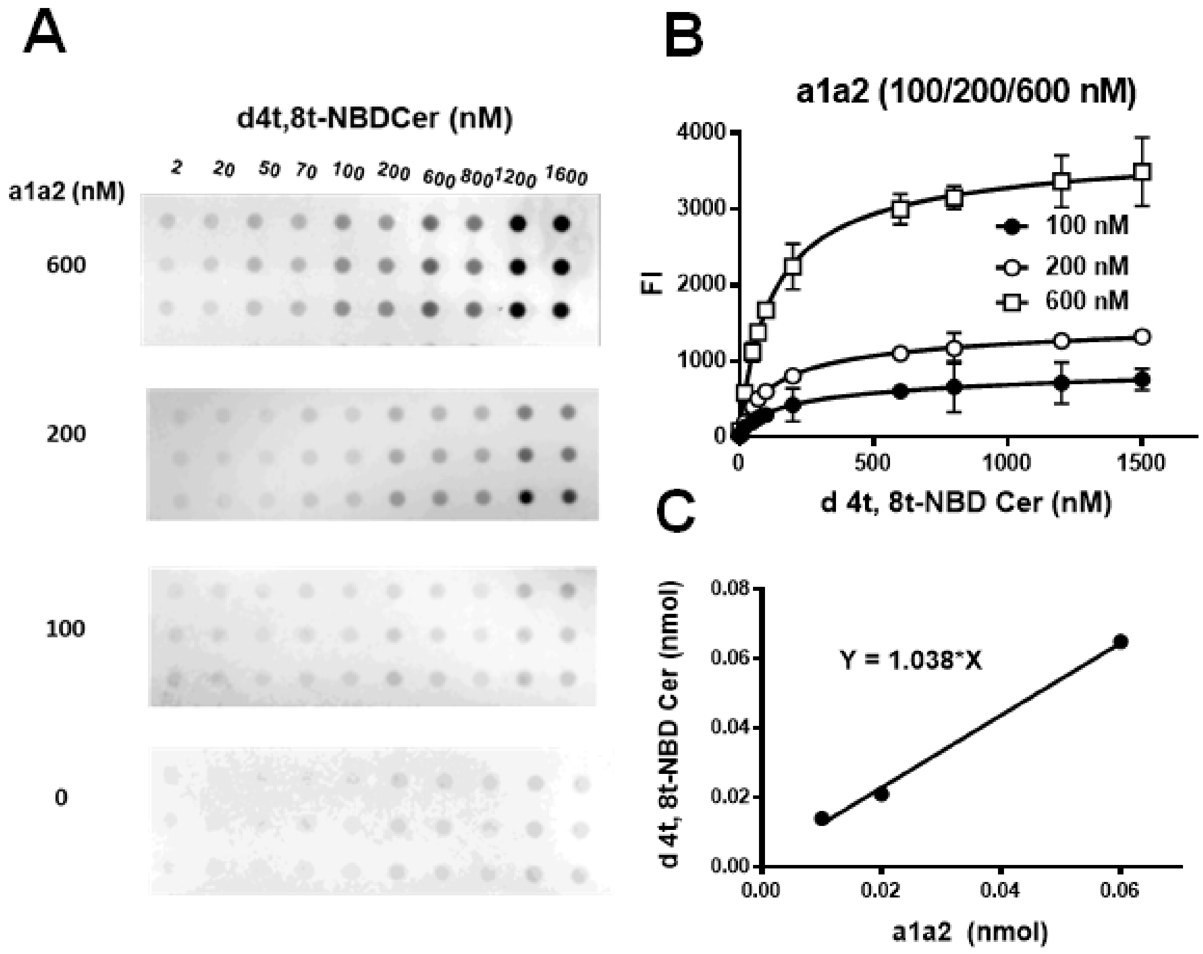

Fig S2

Figure S2: Dot blot analysis of d4t,8t-NBDCer and a1a2.

(A) A dilution series of d4t, 8t-NBDCer (2, 20, 50, 70, 100, 200, 600, 800, 1200, and 1600 nM) was mixed with 100, 200, or 600 nM a1a2, and then subjected to dot-blotting on a nitrocellulose membrane using a dot blot microfiltration apparatus, followed by three washes with TBST buffer.

(B) After FI quantitation of the dotted membranes using a fluorescence imaging instrument, FI was plotted against d4t,8t-NBDCer at 100, 200, and 600 nM concentrations of a1a2. Based on non-linear regression analysis, each point was curve-fitted and connected smoothly by the binding equation (as shown in Fig. 4B).

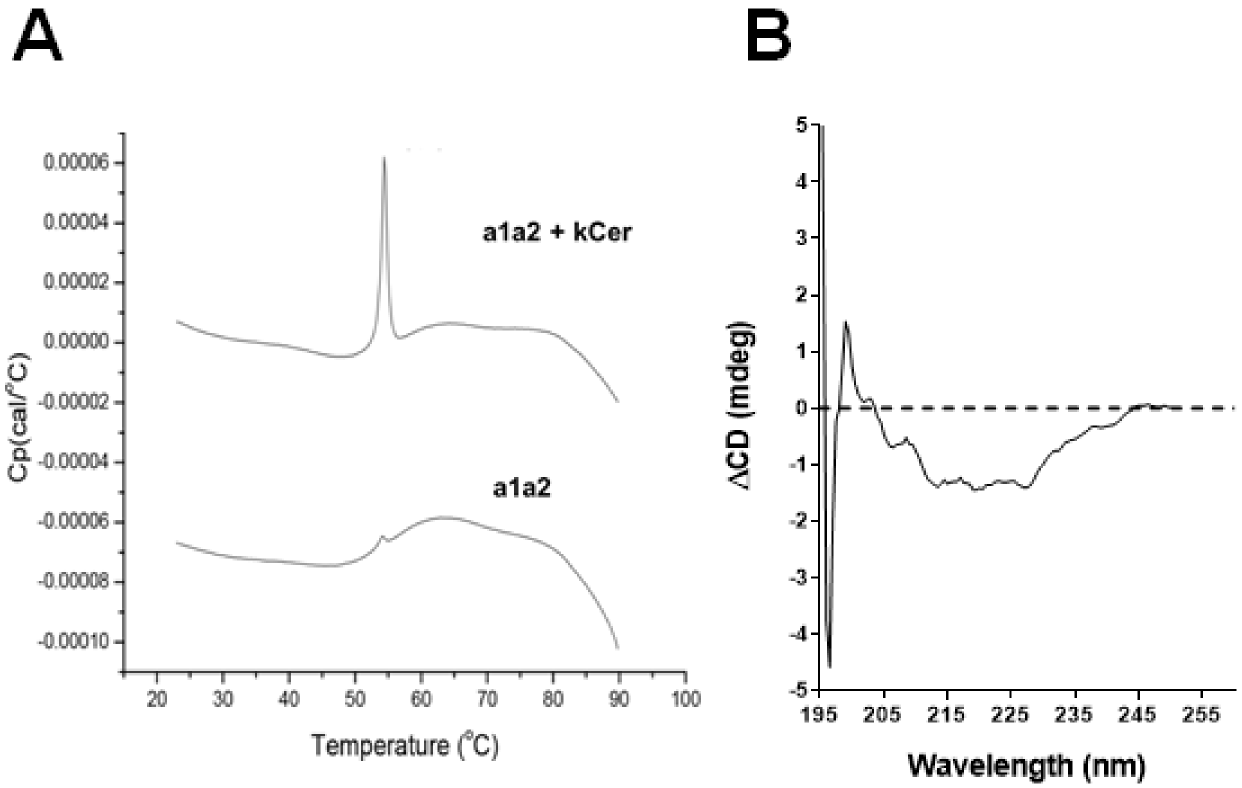

Fig. S3

Figure S3: Interactions between d 4t,8t-C16Cer and the a1a2 domain.

(A) DSC thermograms of the a1a2 protein (3 mg/mL) with and without 100 μM kCer.

(B) CD difference spectrum for a1a2 (0.03 mg/mL) with and without 1 μM kCer.

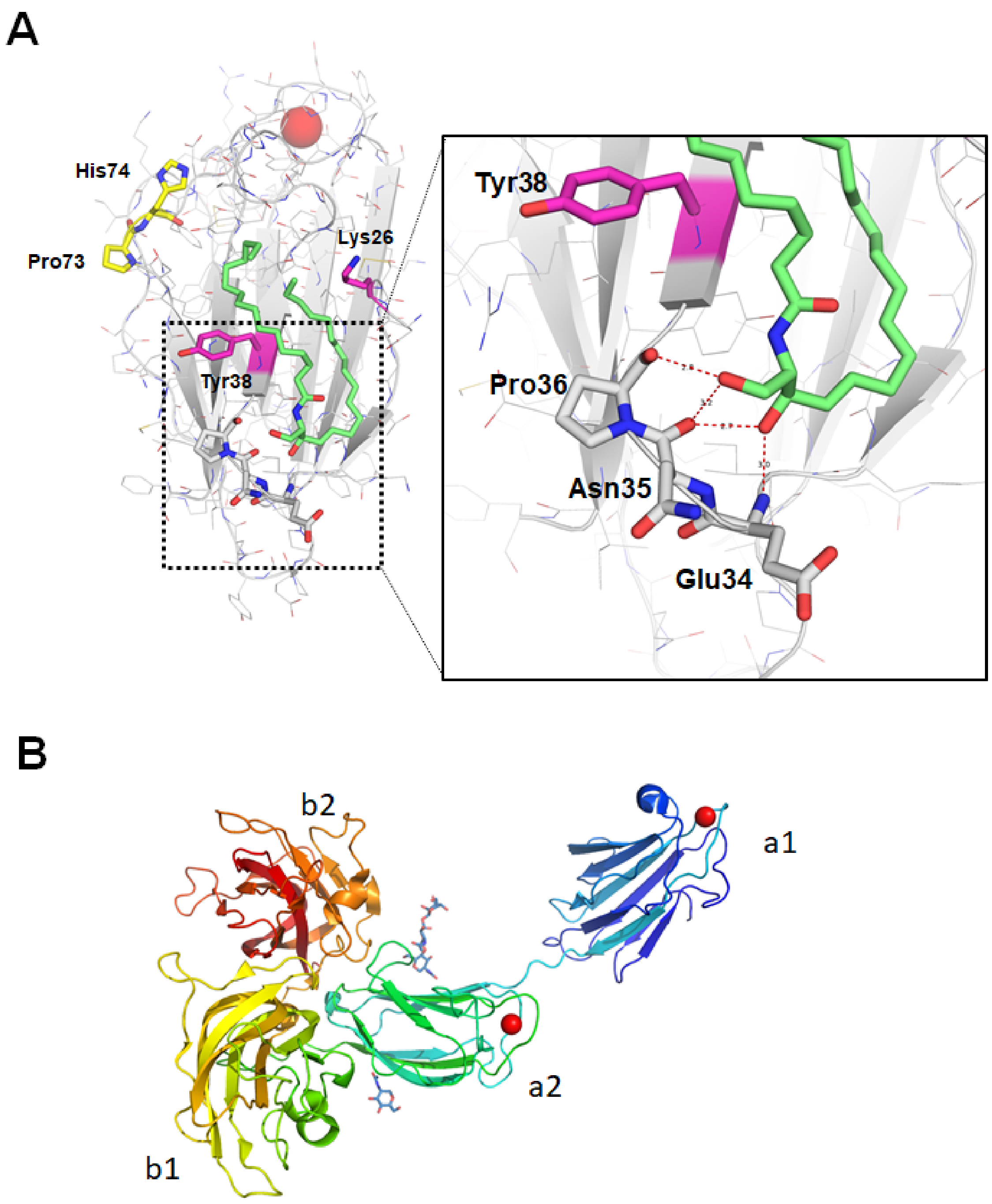

Fig. S4

Figure S4: Molecular docking of d4t,8t-C16Cer and the a1 module at site A.

(A) We propose a hydrophilic interaction between Glu34/Asn35/Pro36 at site A, and the 1- and 3-hydroxyl groups of d4t,8t-sphingadienine of kCer. We also propose a hydrophobic interaction between Tyr38 and Lys26 with palmitic acid and d4t,8t-sphingadienine.

(B) The a1 module is located far away from the a1-b1b2 domain. The image is based on the X-ray crystal structure [27].

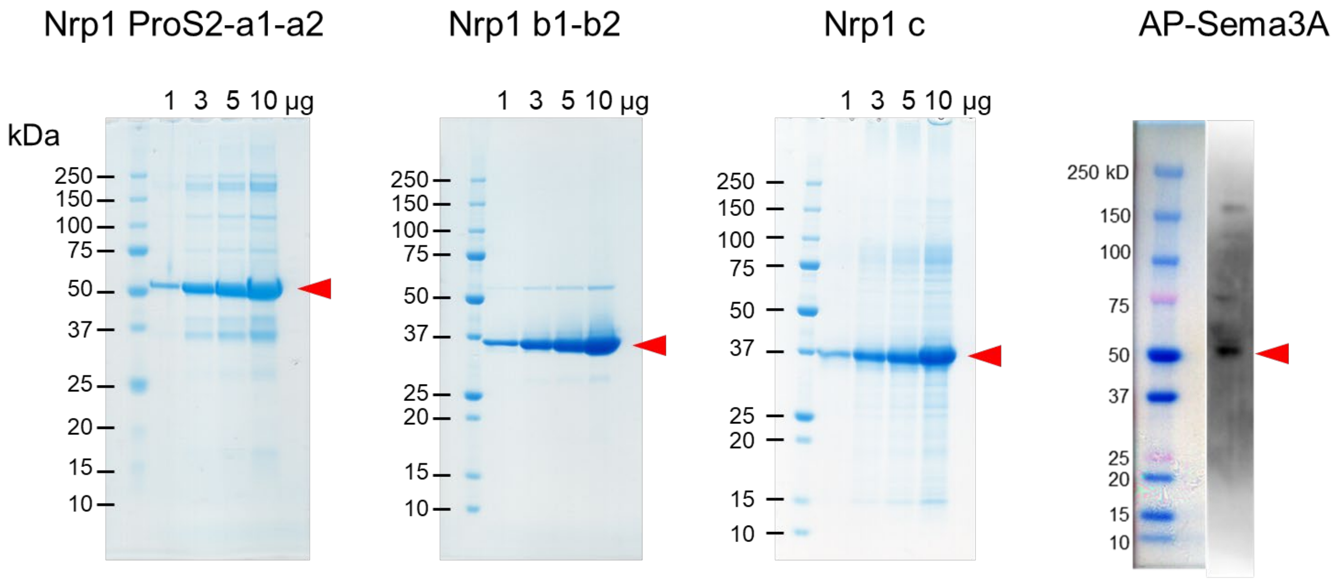

Fig. S5

Figure S5: Purity of the recombinant proteins.

Each of recombinant proteins was tested by SDS-PAGE analysis for Nrp1 domains (a1a2, b1b2, or c) and western blot for AP-Sema3A. by the western blot analysis as described in our previous publication (24). The red arrows show moelcualr weights of the recombinant proteins used for the experiments (Figure 4 and 5), and Each purity of proteins was quantitated by the relative intensities of developed bands using JustTLC system (SWEDAY, Sodra, Sweden). The protein purity was estimated: a1a2 (78.1%), b1b2 (87.2%), c (74.8%), and AP-Sema3A (73.9%).
